# Applications of Remote Sensing to Alien Invasive Plant Studies

**DOI:** 10.3390/s90604869

**Published:** 2009-06-19

**Authors:** Cho-ying Huang, Gregory P. Asner

**Affiliations:** 1 Department of Geomatics, National Cheng Kung University, Tainan 70101, Taiwan; 2 Office of Arid Lands Studies, University of Arizona, Tucson, AZ 85719, USA; 3 Department of Global Ecology, Carnegie Institution for Science, Stanford, CA 94305, USA; E-Mail: gpa@stanford.edu

**Keywords:** biological invasions, high spatial resolution, high temporal resolution, hyperspectral remote sensing, image fusion, light detection and ranging (LiDAR), moderate spatial/spectral resolution

## Abstract

Biological invasions can affect ecosystems across a wide spectrum of bioclimatic conditions. Therefore, it is often important to systematically monitor the spread of species over a broad region. Remote sensing has been an important tool for large-scale ecological studies in the past three decades, but it was not commonly used to study alien invasive plants until the mid 1990s. We synthesize previous research efforts on remote sensing of invasive plants from spatial, temporal and spectral perspectives. We also highlight a recently developed state-of-the-art image fusion technique that integrates passive and active energies concurrently collected by an imaging spectrometer and a scanning-waveform light detection and ranging (LiDAR) system, respectively. This approach provides a means to detect the structure and functional properties of invasive plants of different canopy levels. Finally, we summarize regional studies of biological invasions using remote sensing, discuss the limitations of remote sensing approaches, and highlight current research needs and future directions.

## 1. Introduction

Invasions by alien species (also known as non-native species or non-indigenous species) are among the most formidable of threats to ecosystems and human well-being. Biological invasions have been identified as a major non-climatic driver of global change [1,2]. In the USA, the estimated cost of environmental damages and associated management and control of alien invaders is about $137 billion per year; the total amount could be several times more if monetary values of native species extinctions, biodiversity reduction, ecosystem services and aesthetics can be assessed [3]. Biological invasions may influence the metabolism of ecosystems and can alter disturbance regimes [4]. The ecological effects of alien grass infestation include enhancement of fire frequency and severity, depletion of nutrients, changes in microclimate and alterations in vegetation succession [5]. In addition, invasions can be facilitated by land-use change and other human activities [6,7]. Invasions may even have significant feedbacks with regional and global climate [8]; the rate and spatial pattern of biological invasions (mainly alien plants) may be mediated by other environmental changes such as increased nitrogen (N) deposition and atmospheric CO_2_ concentration, elevated air temperature and variability in precipitation [9-11].

Plant invasions occur across a wide range of bioclimatic conditions. In relatively isolated montane tropical rainforest in Hawaii Volcanoes National Park, the N-fixing tree *Morella faya* (originally from the Azores, Canary Islands and Madeira) invades young volcanic substrates where the growth of native plants (e.g., *Metrosideros polymorpha*) is limited by low N availability [12]. *Morella faya* significantly enhances soil N availability and alters fundamental ecosystem processes [13]. In the Southwest USA, invasions by African grasses have had substantial impacts on drylands during the past century [14]. *Eragrostis lehmanniana*, a tufted perennial South African bunchgrass, and one of the most troublesome species in this arid region, was introduced into Arizona in 1932 to prevent soil erosion and to provide forage for livestock [15,16]. By 1984, *E. lehmanniana* had proliferated throughout 1,450 km^2^ of drylands, yet only half of the area was directly sown [17]. An environmental modeling study indicated that *E. lehmanniana* could potentially inhabit over 70,000 km^2^ in the Southwest USA [18]. In the arctic alpine eco-region of Alaska, despite harsh climatic conditions and low densities of human population and anthropogenic perturbations, 59 non-native plants are recorded and three of them (*Poa pratensis*, *Trifolium hybridum*, *Trifolium repens*) are widely distributed and considered highly invasive [19].

Remote sensing aims to extract information about the condition and/or state of an object without requiring physical contact [20,21]. Sensors can be installed on the ground for detailed, local-scale observations, but in most cases, they are mounted on an airborne or orbital platform to view regions of the Earth's surface. Two of the most important characteristics describing the functionality of a sensor are resolution and energy source. For the purpose of this article, we limit our discussion to sensors having one viewing angle, and with either passive (solar illuminated) or active (e.g., laser pulses, radar) energy sources.

We first introduce studies that delineate the presence of alien plant habitats using moderate spatial/spectral and high spatial resolution remote sensing. We then describe techniques to investigate the phenology (life cycle) of invasive alien plants and/or studies that estimate the presence and abundance of invasive plants via high temporal resolution remote sensing. Hyperspectral remote sensing studies are presented to highlight how they derive chemical properties of non-native plants, followed by active remote sensing that reveals the vegetation three-dimensional (3-D) structure, and multi-dimensional analysis that combines the strengths of different types of remotely sensed data via image fusion. Finally, we discuss the limitations of remote sensing for the regional-scale monitoring, and highlight current research needs and future directions to decipher the complexity of alien plant invasions.

## 2. Moderate Spatial/Spectral Resolution Remote Sensing

The general description of moderate spatial/spectral resolution remote sensing involves data collection at a ground sampling interval (GSI) of 10-100 m in less than 20 channels (bands). Imagery such as from Landsat Thematic Mapper (TM)/Enhanced Thematic Mapper Plus (ETM+), Satellite Pour l'Observation de la Terre (SPOT) and Advanced Spaceborne Thermal Emission and Reflection Radiometer (ASTER) have been heavily utilized in studies of terrestrial vegetation. However, alien plants are often obscured in a backdrop of natural vegetation, and are thus difficult to discern using moderate spatial/spectral resolution images. We found a very limited amount of literature utilizing moderate resolution imagery to observe non-native plants; possibly the only way to make this type of data effective is to detect large stands and patches during the right phenological time for the species of concern. For example, Resasco *et al.* [22] used Landsat TM and ETM+ images acquired in late fall to map an Asian understory shrub *Lonicera maackii* in deciduous forests in Ohio, USA when overstory trees were leafless. Peterson [23] combined field survey data and multi-seasonal ETM+ images to estimate an annual Eurasian grass *Bromus tectorum* in the Great Basin, USA using a specific regression model; the images were collected during the green (late April-early May) and senescent (late June, while native grasses were still green) time periods in *B. tectorum*-infested grasslands. Groeneveld and Watson [24] used the late fall Landsat TM near-infrared band to map the branches of a Eurasian riparian tree *Tamarix ramosissima* in Colorado, USA when the plants were leafless. Despite these successes, in most cases, observing alien plants requires data collected from sensors pushing the limits of at least one type of resolution (spatial, temporal or spectral resolution) since the profiles of these species may be quite similar to those of native plants, from a remote sensing perspective [25].

## 3. High Spatial Resolution Remote Sensing

The most intuitive and straightforward remote sensing approach for alien plant detection is to use high spatial resolution images to visually inspect the spatial distribution of non-native species. The idea in this approach is to pinpoint these species based on their unique spatial patterns or phenological characteristics. Everitt and his colleagues conducted monitoring studies in the USA using aerial photographs taken during the flowering seasons of Eurasian *Euphorbia esula* and Asian *Tamarix chinensis* [26,27]. They found that the visible-wavelength (400-700 nm) reflectance of infested settings was significantly higher due to the bright-colored inflorescence. Color infrared (CIR) aerial photographs are also commonly employed, with resolutions ranging from a few centimeters (aerial videography) to ∼2 m (US National Agriculture Imagery Program; URL: http://www.fsa.usda.gov). Müllerová *et al.* [28] used time-series panchromatic and CIR aerial photographs acquired during the onset of *Heracleum mantegazzianum* invasion (the largest central European forb native to the western Caucasus) in Czech Republic. The brightness of *H. mantegazzianum* was much higher than neighboring vegetation, not only during the flowering, but in the early fruiting season (June-August) due to its distinct structure of fruiting umbels. Distinct fruiting colors afforded an even wider window of opportunity for invasive plant detection, and increased the possibility of monitoring the invasion through time using multiple photographs.

The timing of data acquisition is crucial for aerial photograph analysis [29] since the acquisition is usually made by request; the data may only be useful if collected when the targeted alien plant is distinct from its background and neighboring areas. This issue can be resolved by utilizing multi-spectral high spatial resolution satellite images. Fuller [30] utilized 4-m multispectral IKONOS imagery (GeoEye, Dulles, Virginia, USA) to map an Australian tree *Melaleuca quinquenervia* in south Florida, USA using a back-propagation neural network classifier. Coupling the classification with a landscape fragmentation analysis technique, he found that the spatial pattern of *Melaleuca* was highly aggregated and regularly shaped comparing to the nearby patches of woody plants, which were likely associated with infrastructural development. However, the analysis pointed out that the 4-m GSI of IKONOS multi-spectral bands was insufficient to identify the alien tree at low cover levels (< 50%). Sanchez-Flores *et al.* [31] used pan-sharpened 1-m IKONOS imagery in concert with a genetic algorithm to predict the existence of African grasses *Schismus arabicus* and *Brassica tournefortii* in the Sonoran Desert of Mexico. Laba *et al.* [32] applied a maximum-likelihood classification on QuickBird, another multi-spectral satellite system (DigitalGlobe, Longmont, Colorado, USA) with even finer spatial resolution (2.4 m), to estimate the presence of multiple alien plants (*Lythrum salicaria*, *Phragmites australis* and *Trapa natans*) in diverse tidal wetlands of the Hudson River National Estuarine Research Reserve, USA. They demonstrated that QuickBird was a relatively reliable data source for wetland non-native plant mapping (accuracy assessment ≥ 65%). QuickBird data also proved highly accurate (accuracy assessment ≥ 86%) in delineating an Indian grass *Arundo donax* along a riparian zone in Texas [33].

## 4. High Temporal Resolution Remote Sensing

A potentially severe effect of biological invasion is a decrease in native biodiversity, resulting in significant ecosystem vulnerability [14,34]. Native vegetation with different species and life forms can coexist with different rooting depths and growing seasons [35]; however, the vegetation dynamics of an infested setting may be entirely dictated by the growth of the invading species. Therefore, biodiversity could significantly influence vegetation phenologies.

Studies have demonstrated that it may be possible to monitor vegetation dynamics by using vegetation index time-series data derived from inexpensive, large footprint and high temporal resolution satellite data [36,37]. A vegetation index, such as the commonly used the Normalized Difference Vegetation Index (NDVI) [38], enhances the signal of photosynthetically active vegetation with a combination of visible and near-infrared spectral bands. Several phenological metrics such as vegetation onset time, end time, duration of growth, and rate of vegetation green-up and senescence can be derived from NDVI time-series data [39]. Using the NOAA Advanced Very High Resolution Radiometer (AVHRR), Bradley and Mustard [40] showed that the greenness of the highly invasive grass *Bromus tectorum* was amplified by rainfall, and thus it is possible to locate grass infestations areas in the Great Basin based on its unique phenological response to precipitation. Morisette *et al.* [41] applied a logistic regression to integrate the MODIS (the MODerate resolution Imaging Spectroradiometer) NDVI and Enhanced Vegetation Index (EVI [42]), MODIS derived land cover [43], and thousands of field points to predict the habitat suitability for non-native *Tamarix* spp. in the conterminous USA. The model prediction indicated that, in general, the Southwest (the five most suitable states in order were Arizona, Nevada, New Mexico, Texas and Utah) was the most suitable region for these Asian trees/shrubs. Anderson *et al.* [44] implemented a similar approach to integrate MODIS NDVI into a niche-based model to predict the regional colonization potential of *Lythrum salicaria*, an Eurasia herbaceous, perennial wetland plant in Kansas, USA.

**Figure 1.**
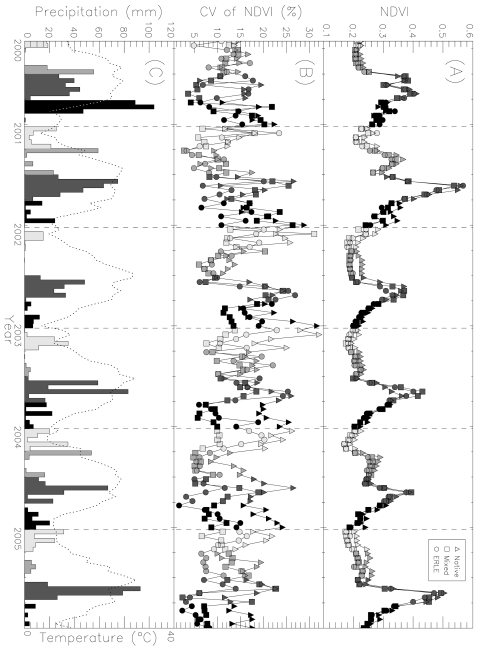
(A) The Normalized Difference Vegetation Index (NDVI) time-series data for Native, Mixed, and *Eragrostis lehmanniana* (ERLE) sites from 2000 to 2005. (B) Spatial variations of NDVI through time among sites demonstrated using the coefficient of variation (CV). (C) Bi-weekly precipitation (bars) and temperature (dotted line) data based upon daily weather records from five stations at the desert grasslands of southern Arizona, USA. Months are evenly divided by four intervals with colors from bright to dark. This figure is adapted from Huang *et al.* [45] with permission from Taylor & Francis.

**Figure 2.**
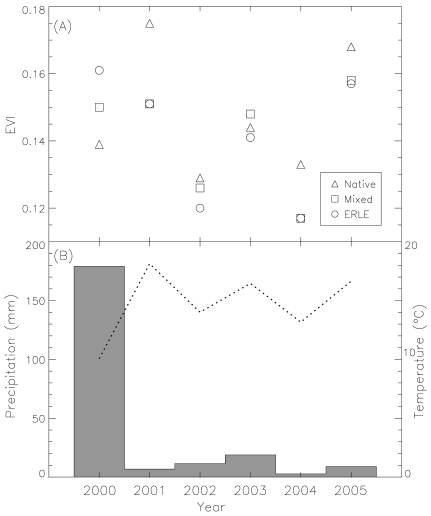
(A) The Enhanced Vegetation Index (EVI) in October (the Moderate Resolution Imaging Spectroradiometer [MODIS] period 19 and 20) of the extreme wet year (2000) and normal years (2001-2005) for field sites in a semi-arid environment of southern Arizona, USA across a gradient *Eragrostis lehmanniana* invasion (native grasslands [Native], a mixture of *E. lehmanniana* and native grasses [Mixed], *E. lehmanniana* invaded grasslands [ERLE]). (B) Mean October temperature (dashed line) and precipitation (gray bars) from 2000 to 2005 of the study region. This figure is adapted from Huang and Geiger [46] with permission from Wiley-Blackwell.

Huang *et al.* [45] investigated the phenologies of semi-desert grasslands in southern Arizona across a gradient of invasions by a non-native perennial grass *E. lehmanniana* using MODIS NDVI and its brightness bands (red and near-infrared reflectance). They found that *E. lehmanniana* invasion altered ecosystem-level phenology in the semi-arid grasslands, which can be clearly delineated in MODIS NDVI and/or brightness time-series data using statistical analyses (Figure 1). Huang and Geiger [46] also found that *E. lehmanniana*-invaded vegetation can produce substantial amount of new tissues during the wet, cool non-growing seasons in semi-desert grasslands as long as there is a sufficient amount of solar radiation (Figure 2). Hence, additional green vegetation signals measured by MODIS EVI during that period may indicate not only the presence but abundance (aboveground biomass) of *E. lehmanniana*. Results indicated that there was a significantly positive relationship between remote sensing predictions and field observations, which may allow us to map *E. lehmanniana* abundance regionally in the semi-desert grasslands.

## 5. Hyperspectral Remote Sensing

Imaging spectrometers (also called hyperspectral imagers) collect a continuous spectrum across a wide region of the visible and/or shortwave region, often using hundreds of spectral bands with a narrow spectral interval (≤ 10 nm). Some of the most commonly used sensors for scientific and management purposes include the Airborne Visible/Infrared Imaging Spectrometer (AVIRIS) [47], Compact Airborne Spectrographic Imager (CASI) [48] and HyMAP [49]. The full optical range of spaceborne hyperspectral data were not available until the launch of Hyperion [50] carried by the Earth Observing-1 (EO-1) satellite. Although the history of hyperspectral remote sensing is relatively short (< 30 years [51]) compared to other types of remote sensing (e.g., aerial photographs > 100 years; satellite images ∼50 years), hyperspectral images are currently the most heavily used imaging source for studies of alien plants.

The main advantages of utilizing hyperspectral data are that detailed spectral profiles can be developed for native and non-native plants, and that specific spectral regions can be analyzed that are most sensitive to the abundance of the species of interest [52]. Williams and Hunt [53] applied a specialized spectral mixture analysis (SMA) to enhance the spectral signals of a European herbaceous perennial plant *Euphorbia esula* from AVIRIS images of the Great Plains, USA. The result was correlated with field data, but the model agreement was also influenced by topography and vegetation types. With the enhancement of remote sensing data acquisition and processing procedures (e.g., see details of AVIRIS instruments, platforms and pre-processing procedures: URL: http://aviris.jpl.nasa.gov/), a hyperspectral sensor can now acquire images with a very fine GSI flown at low altitude. For instance, researchers detected European herbaceous plants (*E. esula* and *Cardaria dra*) in low density (∼ 10%) settings in Idaho, USA [54,55], and precisely identified several riparian and aquatic weeds in Northern California, USA [56,57] using the HyMAP (GSI ≤ 3.5 m) sensor. Miao *et al.* [58] applied SMA to extract signals of an annual Eurasian weed *Centaurea solstitialis* in Northern California from CASI-2 (GSI = 3 m) selected bands by referring to data dimension reduction analyses.

Uses of hyperspectral data are not limited to studies of the presence and/or abundance of alien species, but can be further extended to study the invasion-induced alteration of nutrient fluxes in ecosystems, and also the relationships between alien and native plant spectral profiles and leaf pigment, nutrient and structural properties. Asner and Vitousek [59] combined AVIRIS measures and photon transport modeling to investigate how non-native N-fixing tree *M. faya* and Himalayan understory herb *Hedychium gardnerianum* altered the chemistry of forest canopies across a Hawaiian montane rain forest landscape. They found that *M. faya* significantly increased canopy N concentrations and water content in invaded sites. In contrast, *H. gardnerianum* substantially reduced N concentrations in the overstory tree canopies and enhanced aboveground water content. Once *M. faya* dominated a system, the species also affected nitrification and N-oxide emissions [60]. Asner *et al.* [61] further studied the uniqueness of hyperspectral signatures of 43 Hawaiian native and alien (invasive and non-invasive) trees. They found that: (*i*) Non-native N-fixing trees were spectrally unique from other groups of non-fixing trees; (*ii*) there were detectable spectral differences in highly invasive trees in comparison to other introduced plants; and (*iii*) there were relationships between the observed differences in canopy spectral signatures and relative differences in measured leaf pigments, nutrients, and structural and biophysical properties. They concluded that it is possible to utilize the full AVIRIS spectrum to differentiate these three Hawaiian tree groups, which they exercised in a subsequent operational mapping study [62].

Hyperspectral images from the spaceborne Hyperion sensor are less commonly used for alien species mapping, probably due to the coarse GSI (30 m) and low signal-to-noise ratio compared to airborne imaging spectrometer data [63,64]. Nevertheless, Hyperion provides an opportunity for systematic data collection over a remote region on a stable platform (EO-1 satellite) and at much lower cost than many airborne mapping efforts. The data have been utilized to map coastal alien plants, with demonstrably satisfactory accuracies (overall accuracy ≥ 81%) [65,66]. Asner *et al.* [11] compiled a time-series of Hyperion hyperspectral metrics computed and combined them with field measurements, to measure and compare the structural, biochemical, and physiological characteristics of the highly invasive *M. faya* and common native *M. polymorpha* tree canopies in Hawaiian montane rainforests. They found that the maximum biochemical and physiological differences were measured during hotter and drier periods, with the invader growing faster during these bioclimatically stressful times of the year.

## 6. LiDAR and Image Fusion

Researchers increasingly acquire land surface information by fusing remotely sensed data obtained from two or more sensors, each with different strengths. For example, Walsh *et al.* [67] combined information derived from spaceborne high spatial resolution QuickBird and hyperspectral Hyperion using image classification, object-based image analysis (for QuickBird) or SMA (for Hyperion) to map tropical American guava (*Psidium guajava*; also called common guava) on Isabela Island in the Galapagos archipelago.

LiDAR (Light Detection And Ranging) is an optical remote sensing technology (900-1,064 nm for terrestrial applications) measuring the distance between the sensor and a target surface, obtained by determining the elapsed time between the emission of a short duration laser pulse and the arrival of the reflection of that pulse at the LiDAR receiver [68]. It has been widely used to estimate the three dimensional (3-D) structure (biomass, height, leaf area index [LAI]) of individual plants and vegetation [69]. LiDAR itself is not an efficient method for detecting alien plants because there may not be discernible differences in structure between alien and native canopies; however, LiDAR can be used to monitor the progress of invasion if it is a nearly pure stand [70]. Boelman *et al.* [71] used data collected by AVIRIS and a LiDAR carried by two separate aircraft, along with field-based bioacoustic recordings to study the ecological functioning of native avifauna on slowing *M. faya* trees invasion in native Hawaiian ecosystems. AVIRIS data provided information on *M. faya* presence/absence, whereas the LiDAR data aided in the assessment of forest structural correlates with bird populations.

**Figure 3.**
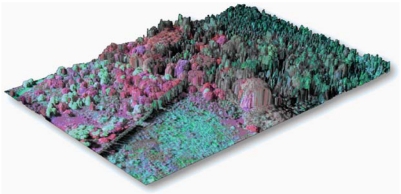
Demonstration of the precision co-alignment and integration of data collected by active (LiDAR) and passive (hyperspectral) remote sensing. This image was collected by the Carnegie Airborne Observatory over a site in Hawaii. The color-coding highlights variation among canopy species and their chemical properties both derived from the hyperspectral data. In this particular example, highly invasive species with unique chemical signatures are shown in red and pink colors, whereas native hardwood forest canopy species are shown in greens and blues. The embedded LiDAR data indicates the height and 3-D structure of each tree crown on the landscape.

**Figure 4.**
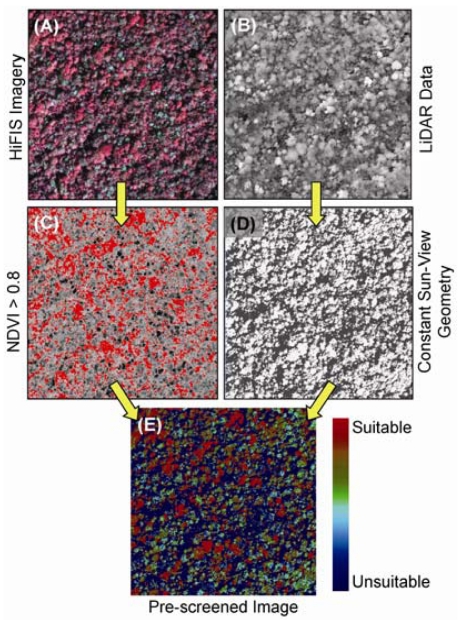
Fully integrated (A) hyperspectral and (B) LiDAR instrumentation provides a means to filter rainforest canopies into comparable units for mapping invasive species. (C) Simple pre-screening of the data based on a minimum NDVI, here set to 0.8, ensures that only foliated canopies are analyzed (red color). (D) Sun-target-view geometry (here, 20°) and minimum canopy height (here, 5 m) is controlled for using the LiDAR data thus pre-screening for view angle effects (white color). (E) Combined, these filters provide a map of canopies suitable for species determinations and comparison. These example images were collected over a Hawaiian rainforest reserve. Adapted from Asner and Martin [77] with permission from Elsevier.

Recently, efforts have been undertaken to fully integrate spectral and LiDAR instrument systems and analytical techniques in order to improve the analysis of vegetation functional and structural properties [72,73]. When instruments are flown on separate aircraft, post-processing steps are required to co-align the data for fusion-based analysis; however, post-flight integration usually results in data misalignments of up to 4 pixels between sensors [74]. While this may be acceptable for some applications [75], a fully-integrated set of measurements, with co-alignment errors ≪ 1 pixel allows for the development and use of fully-automated approaches to measuring canopy chemistry, structure and invasive species occurrence (Figure 3). To our knowledge, one of the only systems in the world with this capability is the Carnegie Airborne Observatory (CAO) [74]. Precisely aligned hyperspectral and LiDAR data afford an opportunity to automatically select and compare vegetation canopy properties: LiDAR can provide a high-resolution canopy surface model, with which spectral data can be selected based on precise calculations of solar position, canopy shape, and viewing geometry [76]. Using this “laser-guided spectroscopy” CAO approach, Asner *et al.* [62,77] were able to map individual crowns of five highly invasive tree species throughout Hawaii Island (Figure 4). Detection errors for most species were only a few percent over more than 221,000 ha of dense rain forest. Once detected with the spectral data, the LiDAR was again used to measure the effects of invasion on the 3-D structure of the canopy. It was shown that different invasive tree species alter the fundamental structure of the forests they invade, but in different ways, either from the ground-up or from the top-down [62].

## 7. The Future of Remote Sensing in Invasive Alien Species Studies

Non-native plant invasions are among of the most disruptive forms of ecological change. Unlike pollution, the proliferation of alien species may not stop even after the source is controlled; unlike the effects of wildfire or logging, invaded vegetation may remain dominated by a single species for a long period of time. Our review suggests that alien plant invasions can be studied using remote sensing when the invader presents a novel structure, phenology or biochemistry relative to neighboring native vegetation. A specific set of remotely sensed data and techniques can be utilized to study each type of invasion characteristic. Table 1 summarizes aspects of biological invasions and the corresponding remote sensing detection strategies. This section focuses on limitations and future directions of remote sensing for alien plant invasion studies.

### 7.1. Limitations of Remote Sensing for Alien Plant Research

As we mentioned earlier, spatial and spectral information provided by moderate spatial and spectral resolution satellite images is insufficient to decipher the complexity of natural environment and further delineate the distribution of alien plants. Although high spatial resolution remote sensing has been proven to be a valid method to map these plants, there are still several drawbacks to these approaches. Aerial photography is one of the most commonly used techniques to map the spatial distribution of alien plants, which involves aircraft scheduling. This would limit the flexibility of data collection. Although this issue can be resolved by utilizing spaceborne high spatial remote sensing, the presence of many non-native species (mainly herbaceous plants) is not discernable even using the newest high spatial resolution sensor GeoEye-1 (GeoEye, Dulles, Virginia, USA), which provides the finest images (panchromatic sensor GSI = 0.41 m; multi-spectral sensor GSI = 1.65 m) from space.

Drake [78] found that even with the finest spatial resolution aerial videography (GSI < 10 cm), it was still a challenge to discern plants at the species level. In addition, phenological changes of vegetation due to the presence of non-native species might not be recognizable if there is no distinct flowering pattern because of the coarse spectral resolution of high spatial resolution images. Visual inspection and photo-interpretation are labor and time intensive, and the quality of the results may be highly dependent upon the skills and experience of the interpreter [79]. Therefore, a high spatial resolution remote sensing approach is less feasible for large-area studies and the quality of the mapping is uncertain and hard to control.

Large swath width and pixel size are unique characteristics of high temporal resolution images (e.g., AVHRR, MODIS). These sensors can frequently monitor the spread of alien plants over a broad region. However, images would capture not only the species but other components such as untargeted plants, surface soils and senescent vegetation, which would limit the ability of images for invasive plant monitoring unless one species dominates an entire system [40,80]. In addition, time-series vegetation data are often contaminated by cloud and snow, and a sophisticated data smoothing algorithm may be required to filter out noise [81]. Large sized plots are usually required to validate the analysis, however, this would make sampling challenging on rough terrain, and the costs for time and labor are very expensive [46]. Moreover, background variation influenced by climate such as soil moisture dynamics could be very significant and overwrites the signals of alien plants.

Data patterns in hyperspectral images can be quite complicated and hard to unravel. In many cases, data reduction techniques are required for generalization and usually involve sophisticated algorithms that “mine” for invasive species signals in the high-dimensional spectral space [e.g., 58]. However, positive empirical results do not necessarily link to clearly identifiable chemical or physiological properties of the plants, which can restrict the use of the techniques to other regions and ecosystems. A spectrometer is usually flown onboard aircraft. Therefore, the swath width of a hyperspectral image is usually narrow, which limits its ability for large-scale invasive plant mapping. Also, scheduling of aircraft and related regulations for different countries could make the data acquisition inflexible. In addition, the robustness of utilizing hyperspectral data to distinguish species types is still uncertain due to the high similarity and variation of spectral signatures among them [82, but see 83].

Although remote sensing data fusion pushes the limits of several aspects of instruments (spatial, spectral and dimensional resolutions) [74], there are still a few inevitable limitations. One major issue is that, due to the nature of data collection expenses (sensors and aircraft rental, labor etc.) and the requirement of high performance computing power [84], the overall cost of this type of research is high and may not affordable for institutions. Also, the sensor integration still does not resolve some of the aforementioned issues in the hyperspectral approaches such as the narrow spatial coverage. Limitations of different types of sensors for non-native plants studies are also summarized in Table 1.

### 7.2. Knowledge Gaps and Research Needs

One type of passive optical sensor is not reviewed in this article was not found in literature with respect to alien invasive species: Multi-angle remote sensing systems that can observe the Earth surface from different angles. Several sensors such as ASTER, AVHRR, MODIS and POLDER (POLarization and Directionality of the Earth's Reflectances) have more than one viewing angle. A sensor specifically designed for observing land surfaces and the atmosphere from different angles is NASA's Multiangle Imaging SpectroRadiometer (MISR), which can provide nine view angles in four optical channels for a targeted area, and GSIs ranging from 275 m (nadir) to 1,100 m (± 70.5°) [85]. This multi-angle viewing function provides a unique opportunity to study the dependence of observed reflectance on solar and sensor geometry relative to the vegetation surface (known as Bidirectional Reflectance Distribution Function [BRDF]). Because the reflectance distribution of vegetation is highly anisotropic, BRDF samples taken from a multi-angular sensor can be used to characterize canopy structure [86,87]. However, there is a knowledge gap in understanding whether the BRDF of canopies changes with biological invasions. For example, vine species *Mikania micrantha* native Central and South American [88] and Asian *Pueraria lobata* [89] have had severe impacts on native forests in Pacific-Asia and in the southeastern USA, respectively. These epiphytic plants climb to the top of native trees, impeding their photosynthetic activity and further “suffocating” the trees. In the invaded ecosystems, the top layer viewed by the sensor might be transformed from tree canopies to these vine species, and thus could be measurable change in the bidirectional reflectance profile of the canopy. Further investigation is required to understand and characterize the ramifications of invasive plants on the BRDF of vegetation.

To date, the CAO system (http://cao.ciw.edu) stretches the limits of modern day optical and active remote sensing techniques. This technology allows us to “CAT scan” terrestrial environments and to diagnose the physiology of terrestrial ecosystems [62]. One important direction for future research would be upscaling these detailed but relatively small scale observations to larger regions via satellite data. Scaling is a method of investigating the ecological patterns and processes at different organizational levels [90,91]. An upscaling (or the bottom-up) procedure usually involves data convolution that degrades the spatial/spectral/dimensional resolutions to match coarse resolution 2-D satellite images [92]. Inevitably, a certain amount of information will be lost through the scaling process. One should know the spatial dependences of data points by computing the influential ranges [93] in order to assess the validity of data generalization [94]. A comprehensive sensitive analysis is often required to determine the propagation of uncertainty across different scales at different resolutions.

Recent efforts attempted to forecast the future distribution of invasive plants by using modeling techniques to predict the suitable (or unsuitable) bioclimatic conditions of invaded regions that are similar (dissimilar) to their origins [18,95]. A straight forward and ecologically sound remote sensing approach may also be applied to diagnose the symptom of pre-invaded vegetation. There could be signs of environmental vulnerability for invasions that are discernible in the spectral/spatial/temporal spaces of remotely sensed data, and these conditions may be utilized for early detection. Results can be validated by comparing with conditions derived from modeling approaches and/or long-term field monitoring records. Remote sensing can also facilitate improved invasive species management. Based on studies reviewed in this article, it appears that differences in the optical characteristics of native and invasive vegetation can be determined. This information may be utilized to evaluate the effectiveness of control techniques to alien invasive plants over large areas. We propose the ideas here; hopefully more research can be conducted in these directions.

## Figures and Tables

**Table 1.** Summary of remote sensing applications for non-native plant studies.

Moderate spatial/ spectral	***Sensor specifications and examples:*** Spatial: 10-100 m. Temporal: Long (16-26 days). Spectral: < 20 bands. ASTER, SPOT, TM/ETM+.
	***Species traits and remote sensing strategies:*** Large stands. Different phenology to co-existing plants. Selection of images acquired in the right season.
	***Limitations:*** Coarse spatial and spectral resolutions unable to extract non-native species from a mixture of different plants.
High spatial	***Sensor specifications and examples:*** Spatial: < 10 m. Temporal: Short (1-4 days). Spectral: ∼5 bands. Aerial photographs, QuickBird, IKONOS.
	***Species traits and remote sensing strategies:*** Unique spatial patterns. Pronounced flowering season. Selection of images acquired in the right season.
	***Limitations:*** Inflexibility of airborne data collection. Pixel spacing still not fine enough to observe plants at the species level. Unable to detect plants with no distinct flowering pattern due to the coarse spectral resolution. Impractical for large scale monitoring due to the time intensive approach (e.g., visual inspection), and small spatial extents.
High temporal	***Sensor specifications and examples:*** Spatial: ≥ 250 m. Temporal: Very short (1-2 days). Spectral: < 40 bands. AVHRR, MODIS.
High temporal	***Species traits and remote sensing strategies:*** Unique phenological characteristics. Combination of models and time-series vegetation indices derived from the images.
	***Limitations:*** Insufficient spectral bands to extract non-native species from large pixels covering other plants, surface soils and senescent vegetation. Time-series vegetation pattern obscured by cloud and snow requiring a statistically sounded smoothing algorithm for noise removal. Difficult to conduct field validation due to the large plot size. Overwriting non-native species signals by climatic variations such as precipitation.
Hyperspectral	***Sensor specifications and examples:*** Spatial: Varied (0.5-30 m). Temporal: Varied. Spectral: > 100 bands. AVIRIS, Hyperion.
	***Species traits and remote sensing strategies:*** Unique signatures in the hyperspectral space. Spectral mixture analysis. Biochemical analysis at the canopy level.
	***Limitations:*** No direct link between invasion mechanism and sophisticate hyperspectral analyses. A small swath width of data collected from aircraft restricting the ability for large spatial scale monitoring. Inflexibility of airborne data collection. High similarity in the spectral space among species.
Active remote sensing	***Sensor specifications and examples:*** Spatial: 0.5-100 m. Temporal: Varied. Spectral: 1 band. 3-D view. LiDAR, RADARSAT.
	***Species traits and remote sensing strategies:*** Large and pure stand. Monitoring the progress of species invasion from data acquired at two time points.
	***Limitations:*** No spectral information and data only useful with good field knowledge.
Image fusion	***Sensor specifications and examples:*** Spatial: ∼0.5 m. Temporal: Varied. Spectral: 200+ bands. 3-D view. Pushing the limits of spatial, spectral and dimensional resolutions in modern remote sensing. CAO.
	***Species traits and remote sensing strategies:*** Unique signatures in the hyperspectral space. Detection of non-native species of different height-levels (overstory and understory) at the very fine spatial scale.
	***Limitations:*** High cost of data collection. Requirement of high performance computing power. Inflexibility of airborne data collection. Small spatial extents restricting very large spatial-scale monitoring.
